# Directional electrodes in deep brain stimulation: Results of a survey by the European Association of Neurosurgical Societies (EANS)

**DOI:** 10.1016/j.bas.2024.102756

**Published:** 2024-02-03

**Authors:** P. Krauss, P. Duarte-Batista, M.G. Hart, J.M. Avecillas-Chasin, M.M. Bercu, V. Hvingelby, F. Massey, L. Ackermans, P.L. Kubben, N.A. van der Gaag, M.T. Krüger, the functional section of the EANS, Olaf E.M.G. Schijns, Tom Theys, Dirk van Roost, Daniel Delev, Kostas Fountas, Karl Rössler, Antonio Goncalves Ferreira, Tipu Aziz, Francois Alesch, Yaroslav Parpaley, Ersoy Kocabicak, Andrey Sitnikov, Oystein Tveiten, Marec von Lehe

**Affiliations:** l Department of Neurosurgery, University hospital Leuven, Belgium; m Department of Neurosurgery, University hospital Ghent, Belgium; n Department of Neurosurgery, University hospital Aachen, Germany; o Department of Neurosurgery, University hospital Larissa, Greece; p Department of Neurosurgery, Medical University of Vienna, Vienna, Austria; q Department of Neurosurgery, Hospital Santa Maria, Lisbon, Portugal; r Department of Neurosurgery, John Radcliffe hospital, Oxford, United Kingdom; s Department of Neuromodulation, Zwickau, Germany; t Department of Neurosurgery, University hospital Bochum, Germany; u Department of Neurosurgery, University Samsun, Turkey; v Department of Neurosurgery, Federal centre of treatment and rehabilitation, Moscow, Russia; w Department of Neurosurgery, Haukeland university hospital, Bergen, Norway; x Department of Neurosurgery, Ruppiner Kliniken, Neuruppin, Germany; a Department of Neurosurgery, University Hospital Augsburg, Augsburg, Germany; b Neurosurgery Department, North Lisbon University Hospital Centre, Lisbon, Portugal; c St George's, University of London & St George's University Hospitals NHS Foundation Trust, Institute of Molecular and Clinical Sciences, Neurosciences Research Centre, Cranmer Terrace, London, United Kingdom; d Department of Neurosurgery. University of Nebraska Medical Center. Omaha, Nebraska, USA; e Department of Pediatric Neurosurgery, Helen DeVos Children's Hospital, Corewell, USA; f Department of Clinical Medicine - Nuclear Medicine and PET Center, Aarhus University, Aarhus, Denmark; g Unit of Neurosurgery, National Hospital of Neurology and Neurosurgery, London, United Kingdom; h Department of Neurosurgery, Maastricht University Medical Center, Maastricht, the Netherlands; i Department of Neurosurgery, Haga Teaching Hospital, The Hague, the Netherlands; j Department of Neurosurgery, Leiden University Medical Center, Leiden, the Netherlands; k Department of Neurosurgery, University Medical Centre Freiburg, Germany

**Keywords:** Deep brain stimulation, DBS, Directionality, dLeads, Steering, Survey, Future technologies

## Abstract

**Introduction:**

Directional Leads (dLeads) represent a new technical tool in Deep Brain Stimulation (DBS), and a rapidly growing population of patients receive dLeads.

**Research question:**

The European Association of Neurosurgical Societies(EANS) functional neurosurgery Task Force on dLeads conducted a survey of DBS specialists in Europe to evaluate their use, applications, advantages, and disadvantages.

**Material and methods:**

EANS functional neurosurgery and European Society for Stereotactic and Functional Neurosurgery (ESSFN) members were asked to complete an online survey with 50 multiple-choice and open questions on their use of dLeads in clinical practice.

**Results:**

Forty-nine respondents from 16 countries participated in the survey (n = 38 neurosurgeons, n = 8 neurologists, n = 3 DBS nurses). Five had not used dLeads. All users reported that dLeads provided an advantage (n = 23 minor, n = 21 major). Most surgeons (n = 35) stated that trajectory planning does not differ when implanting dLeads or conventional leads. Most respondents selected dLeads for the ability to optimize stimulation parameters (n = 41). However, the majority (n = 24), regarded time-consuming programming as the main disadvantage of this technology. Innovations that were highly valued by most participants included full 3T MRI compatibility, remote programming, and closed loop technology.

**Discussion and conclusion:**

Directional leads are widely used by European DBS specialists. Despite challenges with programming time, users report that dLeads have had a positive impact and maintain an optimistic view of future technological advances.

## Highlights

• Comprehensive questionnaire among European functional neurosurgeons.
• State of use of the new directional lead technology in deep brain stimulation (DBS).
• Sentiments and future perspectives of functional neurosurgeons and DBS specialists regarding new DBS technology.

## 1 Introduction

Deep Brain Stimulation (DBS) represented a major advance in stereotactic and functional neurosurgery. The move from lesioning to stimulation provided the option to modulate therapy to optimize beneficial versus adverse effects. Since the 1980s, DBS evolved from experimental rescue therapy to a standard procedure for various conditions, supported by numerous randomised controlled trials, and is now offered by numerous neurosurgical centres in Europe and around the globe (Benabid et al., 1987; Schuepbach et al., 2013; Deuschl et al., 2006; Volkmann et al., 2009; Williams et al., 2010). Despite growing scientific evidence on efficacy, different surgical algorithms exist, and substantial technical breakthroughs are rare or are adopted slowly (Krauss et al., 2021). Until the introduction of “directional leads” (dLeads), these implants had not undergone substantial changes since the inception of DBS. The possibility to steer current and further modify the volumes of tissue activated can provide a more favourable profile between benefits and adverse events, and dLeads are increasingly being used (Schüpbach et al., 2017; Pollo et al., 2014). As pioneers of DBS, the European neurosurgical community has a long tradition and expertise in the field but also has a variety of healthcare systems and inhomogeneous conditions (Ringel et al., 2022).

Numerous questions on the current adoption of leads deserve an answer. Should dLeads be used routinely or not, and for what reasons, anatomical targets, and indications? Should the surgical plan or procedure change with their introduction? Is this new technology cost-effective and time efficient? Are dLeads mainly used for research purposes or also in non-academic centres? Are dLeads considered a benefit or a disadvantage, and what new developments are desired by the DBS community?

This survey of European community DBS specialists by the European Association of Neurosurgical Societies (EANS) functional neurosurgery section, aims to elucidate their usage of and experiences with dLeads, as well as explore their perspectives on other innovations.

## 2 Material and methods

All 82 members of the EANS functional neurosurgery section were contacted via email by board members to ask about creating a questionnaire on directional electrodes. Initially, 13 members from 10 countries responded. Ultimately, seven members from six countries finalised the questions in three virtual meetings. An electronic questionnaire using an online service (Google® Forms) was used to conduct the survey. Participants were recruited via email. In October 2021, all members of the Functional Section of the EANS were invited to participate in this survey. In November 2021, all members of the European Society for Stereotactic and Functional Neurosurgery (ESSFN) were also invited to participate. While completing the questionnaire, participants provided informed consent to be included in this publication. Participants included neurosurgeons, neurologists, and specialist nurses. Members from industry were excluded.

### 2.1 Questionnaire

The questionnaire (Supplemental Material 1) consisted of 50 questions in four categories: a) general information (18 questions), b) surgical strategies (11 questions), c) programming strategies (13 questions) and d) future perspectives (8 questions). Questions were either multiple choice with one or several possible answers (44, 88 %) or open questions (6, 12 %).

The questionnaire can be accessed via this link: https://forms.gle/HW73kHkmPKFfeDuN9.

### 2.2 Statistical analysis

Descriptive statistical analysis was done using IBM® SPSS® Statistics v21.

## 3 Results

The online survey was sent to 82 EANS and 312 ESSFN members (with possible dual memberships). Fifty participants replied. One participant was excluded due to duplicate participation to give a total of 49 included participants (Supplemental Material 2). The time to complete the survey was approximately 10 min. The mean answer rate per participant was 80 % ± 16 % of all questions. The mean response rate per question was 80 % ± 30 % (including all optional questions).

### 3.1 General questions

Ninety-six per cent of participants were from Europe (47/49), one from India (2 %), and one from the United States (2 %), (Table 1). Forty-two (86 %) were male, 78 % (38/49) were neurosurgeons, 16 % (8/49) were neurologists and 6 % (3/49) were nurse specialists. Participant age was 40–49 (37 %), followed by 30–39 (33 %), 50–59 (20 %), and above 60 (10 %).

**Table 1:** 

	n (%)
**Total**	49
**Country**	
Europe	47 (95,9)
USA	1 (2,0)
India	1 (2,0)
**Gender**	
Male	42 (85,7)
**Age**	
<30 years	0 (0,0)
30–40 years	16 (32,7)
40–50 years	18 (36,7)
50–60 years	10 (20,4)
>60 years	5 (10,2)
**Specialization**	
Neurosurgeon	38 (77,6)
Neurologist	8 (16,3)
Nurse Specialist	3 (6,1)
**Work environment**	
University hospital	42 (85,7)
Regional hospital	6(12,2)
Private hospital	0 (0,0)
University and regional hospital	1 (2,0)
**Personal experience in DBS**	
≤1 year	1 (2,2)
1–5 years	7 (15,2)
5–10 years	15 (32,6)
10–20 years	13 (28,3)
>20 years	10 (21,7)
Missing	3
**Institutional experience in DBS**	
≤1 year	0 (0,0)
1–5 years	4 (8,2)
5–10 years	6 (12,2)
10–20 years	14 (28,6)
>20 years	25 (51,0)
**Number of annual DBS cases at institution**	
<15	7 (14,6)
15-30	19 (39,6)
30-50	11 (22,9)
50-100	8 (16,7)
>100	3 (6,3)
Missing	1
**Institutions treating:**	
PD	47 (95,9)
ET	46 (93,9)
DYT	45 (91,8)
OCD	22 (44,9)
Pain	22 (44,9)
Epilepsy	20 (40,8)
GTS	9 (18,4)
Depression	8 (16,3)
Schizophrenia	2 (4,1)
Dementia	1 (2,0)
**Institutional experience with dLead**	
Yes	44 (89,8)

dLead – directional lead; DYT – Dystonia; ET – Essential Tremor; GTS – Gilles de la Tourette syndrome; OCD – obsessive compulsive disorder; PD – Parkinson's Disease.

#### 3.1.1 Experience

Most participants (86 %) worked in university hospitals, 6 (12 %) in regional hospitals, and one in both (2 %). Most institutions had more than twenty years of experience (51 %), followed by ten to 20 years (29 %), five to ten (12 %) and less than five years (8 %). Of these institutions, 39 % were performing 15–30 new DBS procedures per year, 30–50 (22 %), 50–100 (16 %), <15 (14 %), or >100 (6 %). The specialists themselves had 5–10 years of experience (31 %), followed by 10–20 (27 %), >20 (20 %), 1–5 (14 %), or <1 year (2 %).

#### 3.1.2 Indications

Most centres performed DBS for movement disorders including Parkinson's disease (96 %), tremor (94 %) and dystonia (92 %). Other indications included DBS for pain (45 %), obsessive-compulsive disorder (45 %), and epilepsy (41 %). Less common indications were Gilles de la Tourette syndrome (GTS) (18 %), depression (16 %), schizophrenia (4 %), and dementia (2 %). Ninety per cent of experts (44/49) stated they were implanting dLeads at the time point of the survey.

#### 3.1.3 Systems

Most participants stated that they were using more than one company. Boston Scientific (BSC) was used by the majority (68 %), followed by Medtronic (56 %), and Abbott (49 %). Forty-two per cent preferred the BSC system (21/44). Twenty-five per cent (11/44) stated that their preference would depend on the indication. 18 % (8/44) favoured the Abbott system, and 9 % (4/44) Medtronic.

#### 3.1.4 Reasons and indications to implant dLeads

**Fig. 1:**
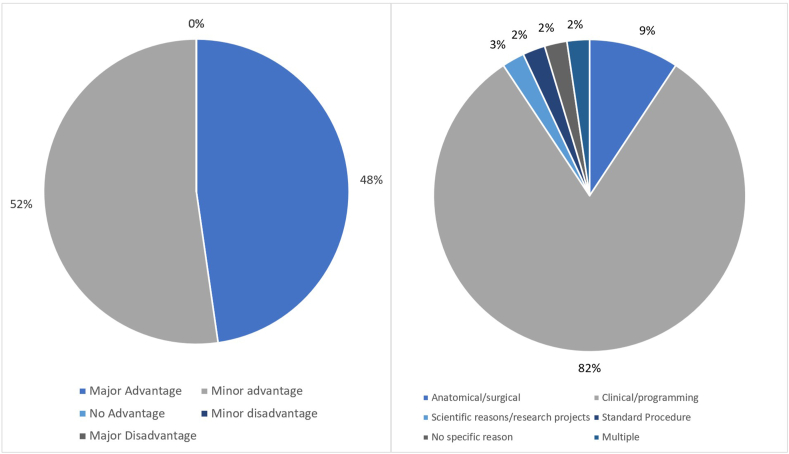
General questions regarding experience with directional leads. Left: What is your personal opinion on dLeads in DBS? (n = 44); Right: What is the main reason to implant a dLead at your institution? (n = 44).

When asked about the main reason dLeads were implanted at their centre (Fig. 1), most cited clinical/programming reasons (80 %). Nine percent had anatomical or surgical reasons. Two percent stated either scientific/research reasons, no specific reason, or that it had become a standard.

Most participants implanted dLeads for PD (98 %), tremor (86 %) and dystonia (82 %). They were much less frequently used for pain (21 %), epilepsy (11 %), obsessive compulsive disorder (OCD) (5 %), and depression (5 %). None of the participants implanted dLeads in patients with GTS, schizophrenia, or dementia.

DLeads were considered a minor advantage by 52 %, and a major advantage by 48 % over standard leads (Fig. 1). No respondents thought that dLeads provided no advantage or were a disadvantage.

### 3.2 Surgery

#### 3.2.1 Planning and implanting leads

**Fig. 2:**
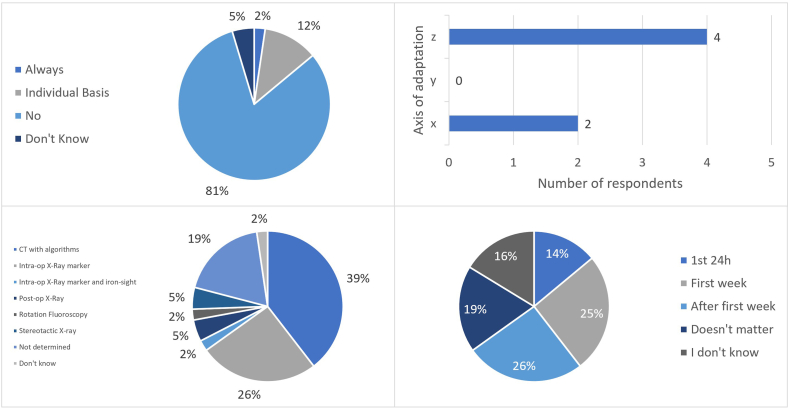
Questions regarding surgical strategies. Upper-left - Does your planning process differ, if implanting dLeads compared to standard electrodes? (n = 43); Upper-right – Type of planning adaptation, according to axis (n = 6); Bottom-left – Strategies to determine the lead's final orientation (Question: How do you determine the final orientation of the electrode?) (n = 43); Bottom-right – Ideal timing to determine final orientation (Question: When do you believe is a good time to determine the final orientation?) (n = 43). CT – Computed Tomography.

Six of 43 respondents (14 %) with dLead experience claimed their planning with dLeads differed when compared to non-directional electrodes (Fig. 2). Five of these respondents changed their plans on an individual patient basis, and only one changed their planning strategy systematically. The most common adjustment was in the z-axis (4 respondents), followed by the x-axis (2 respondents). Two respondents gave a detailed answer regarding their changes, and both stated they ended up planning slightly deeper to leave a segmented contact in the target. Regarding which nucleus needed plan adjustments, all six respondents changed their planning for subthalamic nucleus (STN) and ventral intermediate nucleus of the thalamus (VIM) and four for globus pallidus (GPi). Five out of six respondents agreed that one should not aim for lower side-effect thresholds during intra-operative clinical testing; however, two admitted to accepting this to some degree in practice. One respondent stated that one should aim for lower thresholds.

Based on the survey, 74 % (32/43) of respondents with experience with dLeads attempt to direct the lead in a specific direction during implantation. Of these, 91 % (29/32) place the guide marker facing the anterior direction, normally considered 0° of orientation. Only 3 respondents reported a different strategy: Guide marker straight back (n = 1) and orientation defined individually depending on the target implanted (n = 1) or individual anatomy (n = 1). No respondent reported facing the guide marker lateral- or medially, or based on MER, LFP or intra-operative testing.

#### 3.2.2 Determining the lead's final orientation

Most respondents with dLead experience (79 %: 34/43) reported using some technique to determine the final orientation of the lead (Fig. 2). The two most used techniques were algorithms based on the post-operative CT (50 %: 17/34) and marker identification on the intra-operative X-ray (32 %: 11/34). Other methods used were post-operative X-ray (n = 2), stereotactic X-ray (n = 2), intra-operative X-ray using the guide marker and the iron-sight (Reinacher et al., 2017; Egger et al., 2021) (n = 1), and rotational fluoroscopy (n = 1). Eight respondents (19 %) claimed they do not determine the electrode's final orientation, and one (2 %) did not know how to answer.

As for the best timing for this determination (Fig. 2), six (14 %) respondents thought it should be done within the first 24h, eleven (26 %) within the first week, and eleven (26 %) after the first week. Eight respondents (19 %) considered timing irrelevant, and seven (16 %) had never thought about this issue.

When comparing the determined final orientation with the initially intended orientation, more than a third of respondents (38 %: 16/42) claimed to have good accuracy within acceptable clinical limits set by the team. Almost 12 % of respondents (n = 5) claimed moderate accuracy with a consistent variance. Eight respondents (19 %) claimed variable or poor accuracy.

### 3.3 Programming

Seventy-one percent (31/44) of respondents had experience in DBS programming. In most centres, neurologists performed programming (84 %), followed by DBS nurses (50 %) and neurosurgeons (50 %). Multiple answers were possible; in many centres, all three disciplines are involved in this aspect.

#### 3.3.1 Reasons to use directionality

**Fig. 3:**
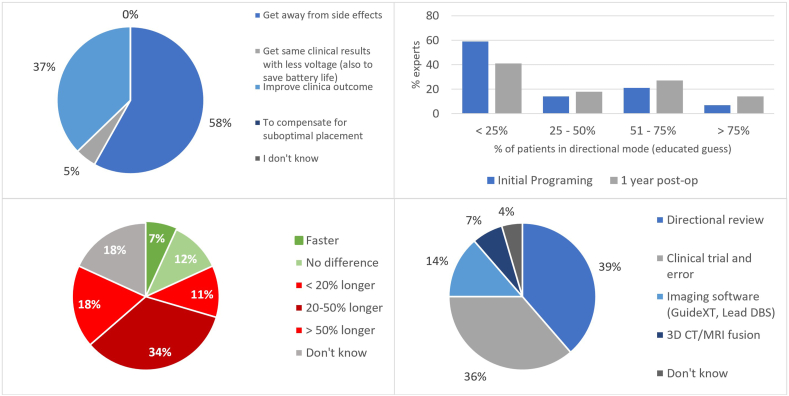
Questions on programming strategies. Upper left – Reasons to use steering (Question: What do you mainly use steering for?) (n = 44); Upper-right – An educated guess given by the experts regarding the percentage of patients starting on a directional mode right after surgery vs. 1 year after surgery (Questions: 1 - How many of your patients do you start on a directional mode up-front (educated guess)?; 2 - How many of your patients are on a directional mode one year after implantation (educated guess)?) (n = 44); Bottom-left – Time taken to program dleads (Question: How much longer do you need to program a dLead as compared to a standard lead?) (n = 44); Bottom-right – programming Method used for dleads (Question: How do you usually do your directional programming?) (n = 44). 3D – three-dimensional, CT – Computed Tomography, MRI – magnetic resonance imaging.

Most participants used directionality to steer away from side effects (57 %, 25/44), 36 % to improve clinical outcomes, and five percent to get the same results with less voltage (Fig. 3).

For initial programming, 32 % start 0 % of their patients on a directional setting. 36 % start <25 % on a directional setting, 14 % start 25–50 %, nine percent start 50–75 % and another nine percent start >75 % on a directional setting. Forty percent of experts stated that after one year, <25 % of patients are on a directional setting, 18 % state 25–50 % are on a directional setting, 27 % have 50–75 %, and 14 % have >75 % on a directional setting.

#### 3.3.2 Programming time and technique

Sixty-three percent of respondents reported dLead programming taking longer, and only seven percent (n = 3) stated they were faster (Fig. 3). When asked how programming was made faster, they said (1): using imaging software, no monopolar review is needed; (2) GuideXT, and (3) it is obvious.

Seventy-five percent of participants still use a non-image-assisted programming method, namely clinical trial and error (39 %) and directional review (36 %) (Fig. 3). Fourteen per cent use imaging software, and seven percent use 3D CT/MR fusion.

#### 3.3.3 Advantages and disadvantages

When asked about the major disadvantage of dLeads, most experts (54.5 %) stated they are too time-consuming to program, and 13.6 % found them too expensive.

When asked if they felt the introduction of directionality had improved clinical outcomes of patients, most stated that they found them to be a bit better (52.5 %), and 12.5 % found they were much better than standard electrodes. Fifteen per cent were unsure, another 15 % found them no better but no worse, and five per cent found them to be a bit worse. Even though dLeads were regarded as mostly beneficial, participants that practice in high volume centres were less enthusiastic about dLeads regarding the overall benefit (Q41: p > .001; r = −0.50) and personal opinion (Q24: p > .001; r = −0.40). This also applied to more experienced DBS specialists (Q24: p = .02; r = −0.36 but not Q:41: p = ns; r = −0.28) (spearman correlation). Age, gender, or the institution's experience with DBS did not influence the opinion towards dLeads.

#### 3.3.4 Most beneficial indications

With regard to indications, PD, ET and dystonia patients were thought to benefit from directionality by 75 %, 48 % and 30 % of respondents, respectively. Fourteen percent thought they were beneficial for patients with pain, and nine percent for patients with epilepsy. Only four percent and two percent suggested dLeads were beneficial for patients with OCD and depression, respectively. Eleven percent stated they would be most beneficial for patients with new indications.

### 3.4 Future of directional stimulation

**Fig. 4:**
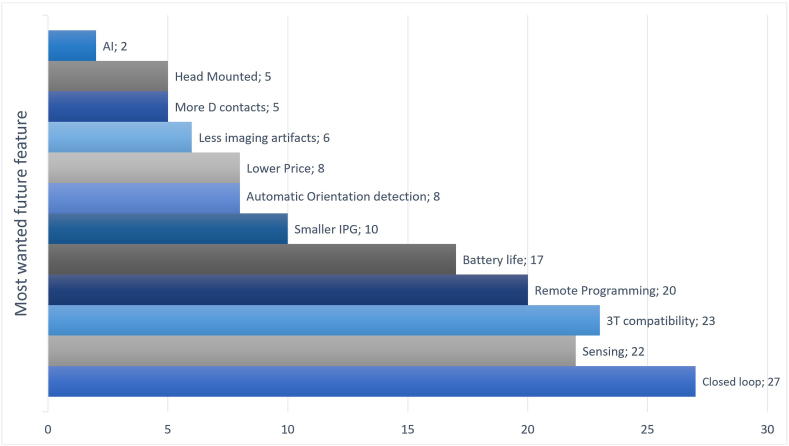
What would be your three most important features of a future DBS system? - Respondents were asked to choose the three most wanted features for a future DBS system. The graphic shows the total number of respondents that choose each feature. AI – Artificial intelligence; IPG – implanted pulse generator.

When asked what features would be most important in a future directional DBS system (Fig. 4), the top responses were: closed-loop stimulation (27/49), 3 T (3T) MRI compatibility (23/49), sensing electrodes (22/49), and the capability for remote programming (20/49). Additional desirable features included: increased IPG life, smaller IPG size, automatic detection of directionality, improved affordability, and reduced artefacts on MRI. More directional contacts and cranial-mounted systems were among the lowest-ranked choices.

#### 3.4.1 Future wishes

When considering contact configuration, 38/47 felt this should change from the currently available 1-3-3-1 configuration. However, only 2/47 felt that more than four directional contact levels would be required. The most desirable configuration was 3-3-3-3 (27/47). A further 7/47 felt that four directional contacts per level would be desirable.

Views on how technological advances would improve patient outcomes were generally optimistic. Only 3/47 felt that sensing would not improve outcomes, 6/48 felt that sensing would not improve the adoption of directional electrodes, and 9/49 did not feel artificial intelligence would improve outcomes. There was a general consensus that most existing systems would evolve to become closed-loop in the future (46/49). However, the application of patient-controlled programming was more contentious, with 14/49 highlighting some reluctance to use it to provide more parameters than in current clinical practice.

## 4 Discussion

This survey of the EANS) aimed to elucidate the usage and experiences with dLeads and explore their perspectives on other innovations. With <16 %, the response rate was lower than reported in other surveys in medical personnel, which might be due to the absence of personalized E-Mail invitations or (financial) incentives. Nevertheless, surveys among medical personnel have been shown to be more robust regarding response bias compared to the general population (Booker et al., 2021; Cunningham et al., 2015; Kellerman and Herold, 2001).

### 4.1 Surgical strategies

Most respondents did not change surgical strategy when implanting directional leads as compared to omnidirectional leads. Those who did change strategy mainly implanted directional leads slightly deeper so that one of the directional levels was at the target, which is in line with the literature when targeting the VIM (Krüger et al., 2021a).

Determining lead orientation was highly inconsistent among respondents. Most preferred using post-operative CT scan or intraoperative X-ray, with very few using other methods (e.g. rotational fluoroscopy) (Reinacher et al., 2017; Hellerbach et al., 2018; Hunsche et al., 2018; Sitz et al., 2017). Large variability was also evident on when to perform images that would reliably determine final orientation. Numerous published papers have addressed this topic (Dembek et al., 2019; Krüger et al., 2021b). In an animal study, twisting the leads resulted in large, delayed rotations (Rau et al., 2021). However, most clinical studies show that potential rotation is minor within the first 24 h and that electrode orientation remains stable thereafter (Krüger et al., 2021b; Dembek et al., 2021; Lange et al., 2021a). The survey results reflect uncertainty about these aspects and the need for further education on the topic.

### 4.2 Programming

Initially, the number of patients programmed on directional settings was stated as relatively low. However, this number increased substantially one year after the initiation of stimulation. This trend is supported by the literature where the number of patients on directional modes varies from 33 % to 85 % for the STN and 39 %–92 % for the VIM, with a trend towards larger numbers on directional modes as time progresses (Veerappan et al., 2021; Rammo et al., 2021; Mishra et al., 2023; Karl et al., 2022; Reker and Steffen, 2020; Zitman et al., 2021; Koivu et al., 2022).

Most experts found dLeads very time-consuming to program due to the large number of programming options. Most stated that they needed 20–50 % longer than with standard leads, a disadvantage often reflected in the literature (Krüger et al., 2021a; Brinke et al., 2018; Debove et al., 2023; Maciel et al., 2021). Only three participants stated that they were faster and explained that they were using image guidance to decrease programming time. Moving forward, more experience using imaging and algorithms to support programming may allow speeding up the process while optimising clinical outcome. The rising number of publications on image- and algorithm-guided programming supports this notion (Lange et al., 2021b; Mei et al., 2022; Wenzel et al., 2021; Roediger et al., 2022, 2023; Waldthaler et al., 2021).

Overall, most experts found the clinical outcome of patients to be slightly or much better. None of them found them to be worse. This positive attitude is only partially reflected by the literature with most papers showing theoretical but no clinical benefits (Rammo et al., 2021; Bruno et al., 2021; Steffen et al., 2020; Shao et al., 2020; Rebelo et al., 2018; Schnitzler et al., 2022; Sabourin et al., 2020; Dembek et al., 2017; Steigerwald et al., 2016) and only very few showing some benefit (Krüger et al., 2021a; Hurt et al., 2023; Pintér et al., 2023). However, most of these studies are on newly implanted patients with short follow-up times, and the discrepancy could well reflect a gap in the literature that may be filled over time.

Most experts believed that mainly patients with well-established indications, such as PD, tremor and dystonia would benefit most from directionality. This is very much in line with the literature so far, where most published studies are on PD and tremor (Bruno et al., 2021; Shao et al., 2020; Rebelo et al., 2018; Schnitzler et al., 2022), with only a small number of case reports or small case series on dystonia, pain or psychiatric diseases (Abel et al., 2021; Asahi et al., 2021; Krüger et al., 2021c; Coenen et al., 2022; Lopez et al., 2021), and no studies on depression or epilepsy.

### 4.3 Future

Many of the top requests for future directional devices are already being implemented, namely 3-T MRI conditionality, sensing, and remote programming. MRI conditionality up to 3T may reflect the need for further imaging in an ageing population with co-morbidities (Hayley et al., 2022) rather than being used for DBS lead localisation due to the significant artefacts involved. However, there is also potential for novel research applications, as an overlap with the management of (complex) epilepsy disorders using a sensing closed-loop stimulation system is being recognized. Implementation of advanced tractography, analysis of continuously recorded electrophysiological variations, and ultimately fusing the data with high resolution anatomical novel scans (such as Fast Gray Matter Acquisition T1 Inversion Recovery, FGATIR) are expected to yield a better understanding of brain connectivity and function, possibly improving therapeutic outcomes (Miao et al., 2022; Gadot et al., 2023). The major request for closed-loop stimulation is being explored but is not yet a feasible option in clinical practice. The desire for increased IPG life and reduced size presumably refers to primary cell systems. Increased device longevity and improved cost-effectiveness would help improve access at a global level. Interestingly, the demand for other technologies, such as increased contact numbers and cranial-mounted devices, was low, suggesting a more pragmatic approach within this group of experts. Great hope was placed in closed-loop and artificial intelligence; however, it is vital that neurosurgeons are engaged in directing their application to address clinically meaningful problems. Along similar lines, the enthusiasm for sensing was mainly fuelled by the desire to address programming complexity, whereas much of the basic science that underpins sensing is currently not focused on this issue. Whether these new technologies condense in substantial benefits needs to be further addressed in larger studies that investigate the effects on clinical outcome and cost effectiveness of such more complex technologies.

## 5 Conclusion

Directional leads are a well-adopted technology among European DBS specialists. These experts have an optimistic view of future technological advances and have embraced directional technology for the foreseeable future. Nevertheless, more publications on directional electrodes, with larger numbers of patients and longer follow-up times, are required to support this overall positive attitude.

## Funding

This research did not receive any specific grant from funding agencies in the public, commercial, or not-for-profit sectors. This research did not receive any specific grant from funding agencies in the public, commercial, or not-for-profit sectors.

## Declaration of competing interest

PK has received travel funding from Abbott and Boston Scientific and has received speaker honorary from 10.13039/501100002806Carl Zeiss Meditec AG. PDB has received travel funding from Medtronic and Boston Scientific.

MGH, JMAC, MMB, VH, FM, VH, LA, PLK, NAvdG have nothing to declare. MTK is a consultant for Boston Scientific and Brainlab and has received travel funding and consultancy fees from Elekta and Medtronic.
